# Transcriptome Analysis of Buds and Leaves Using 454 Pyrosequencing to Discover Genes Associated with the Biosynthesis of Active Ingredients in *Lonicera japonica* Thunb.

**DOI:** 10.1371/journal.pone.0062922

**Published:** 2013-04-25

**Authors:** Liu He, Xiaolan Xu, Ying Li, Chunfang Li, Yingjie Zhu, Haixia Yan, Zhiying Sun, Chao Sun, Jingyuan Song, Yu’an Bi, Juan Shen, Ruiyang Cheng, Zhenzhong Wang, Wei Xiao, Shilin Chen

**Affiliations:** 1 Institute of Medicinal Plant Development (IMPLAD), Chinese Academy of Medical Sciences and Peking Union Medical College, Beijing, China; 2 State Key Laboratory of New-tech for Chinese Medicine Pharmaceutical Process; Jiangsu Kanion Pharmaceutical Co. LTD, Lianyungang, China; 3 Beijing University of Chinese Medicine, Beijing, China; 4 Institute of Chinese Materia Medica, Academy of Chinese Medical Sciences, Beijing, China; Cankiri Karatekin University, Turkey

## Abstract

**Background:**

*Lonicera japonica* Thunb. is a plant used in traditional Chinese medicine known for its anti-inflammatory, anti-oxidative, anti-carcinogenic, and antiviral pharmacological properties. The major active secondary metabolites of this plant are chlorogenic acid (CGA) and luteoloside. While the biosynthetic pathways of these metabolites are relatively well known, the genetic information available for this species, especially the biosynthetic pathways of its active ingredients, is limited.

**Methodology/Principal Findings:**

We obtained one million reads (average length of 400 bp) in a whole sequence run using a Roche/454 GS FLX titanium platform. Altogether, 85.69% of the unigenes covering the entire life cycle of the plant were annotated and 325 unigenes were assigned to secondary metabolic pathways. Moreover, 2039 unigenes were predicted as transcription factors. Nearly all of the possible enzymes involved in the biosynthesis of CGA and luteoloside were discovered in *L. japonica*. Three hydroxycinnamoyl transferase genes, including two hydroxycinnamoyl-CoA quinate hydroxycinnamoyl transferase genes and one hydroxycinnamoyl-CoA shikimate/quinate hydroxycinnamoyl transferase (HCT) gene featuring high similarity to known genes from other species, were cloned. The *HCT* gene was discovered for the first time in *L. japonica*. In addition, 188 candidate cytochrome P450 unigenes and 245 glycosyltransferase unigenes were found in the expressed sequence tag (EST) dataset.

**Conclusion:**

This study provides a high quality EST database for *L. japonica* by 454 pyrosequencing. Based on the EST annotation, a set of putative genes involved in CGA and luteoloside biosynthetic pathways were discovered. The database serves as an important source of public information on genetic markers, gene expression, genomics, and functional genomics in *L. japonica.*

## Introduction

*Lonicera japonica* Thunb. is a perennial, evergreen, twining vine. It has double-tongued flowers that open white and fade to yellow. This plant is called Jinyinhua (literally “gold silver flower”) in Chinese and is cultivated worldwide as an ornamental plant because of its numerous sweet smelling flowers. However, *L. japonica* is believed to be invasive to the ecology of some American countries because of its strong viability [1]. It is widely cultivated in China as well as in other Asian countries, such as Japan and Korea, as a commercially valuable plant. Flos Lonicerae Japonicae (FLJ), the dried bud of the *L. japonica,* has been used for thousands of years in Chinese medicine for its antipyretic, antidotal, and anti-inflammatory properties. It has been recorded in the “Ben Cao Gang Mu” (Compendium of Materia Medica) as early as the 17^th^ century. Thus far, FLJ is a popular drug for the treatment of influenza virus. Since the outbreak of SARS and avian influenza viruses in China, the use of *L. japonica* has significantly increased.

Several studies have investigated *L. japonica* to improve its applications and a large number of active ingredients have been extracted from the plant, including phenolic acids [2]–[4], flavones [5]–[8], triterpenoid saponins [9]–[13] and volatile oils [14], [15]. These ingredients mediate multiple properties, including antioxidant [16]–[18], anti-inflammatory [19], anti-carcinogenic [20]–[26], and antiviral [27], [28] effects. Among these ingredients, chlorogenic acid (CGA) and luteoloside are the primary active components that have attracted the most attention from researchers. The contents of these two components in *L. japonica* are the main valuation criteria of this plant.

CGA was first found in sunflower seeds [29] and is an important phenolic acid that comes from secondary metabolism pathways in many plants [30], [31]. CGA is often used in medicines and foods because of its high anti-oxidative activity [32]–[34].The biosynthetic pathway of CGA is controversial, although three metabolic pathways have been postulated based on previous research. The first mechanism indicates that CGA is produced from quinic acid and caffeoyl CoA and catalyzed by the hydroxycinnamoyl-CoA quinate hydroxycinnamoyl transferase (HQT) [35]–[39]. The second mechanism indicates that CGA comes from quinic acid and caffeoyl-D-glucose and is catalyzed by hydroxycinnamoyl D-glucose: quinate hydroxycinnamoyl transferase (HCGQT) [40]–[42]. The third mechanism proposes that CGA comes from p-coumaroyl quinic acid and is catalyzed by hydroxycinnamoyl CoA shikimate/quinate hydroxycinnamoyl transferase (HCT) [43], [44]. Recent research has demonstrated that HQT is an indispensable enzyme in the synthesis of CGA in *L. japonica* [38].

Luteoloside belongs to a group of natural flavonoids and exists in many plants. It comes from luteolin by 7-O-beta-glucosyltransferase, which transfers the glucosyl moiety to the 7-O-position of the substrate [45]–[50]. The biosynthetic pathway of luteolin is clear in other species [51] but all of the genes involved in the biosynthesis of luteolin in *L. japonica* have not yet been found. *L. japonica* is a very important plant material in the study of CGA and luteoloside because of their high content in this species.

Limited genomic and transcriptomic information is available for *L. japonica* in GenBank (September 2012). The 100 million sequence reads obtained from *L. japonica* and *L. japonica* Thunb. var. chinensis (Watts) using Illumina Genome Analyzer II and the approximately 6000 expressed gene tags for each of the three flower development stages from FLJ are not available in NCBI [52]. In addition, most enzymes involved in the biosynthetic pathways of active compounds have not been reported.

Besides Illumina GA II, the Roche/454 GS FLX platform, another high-throughput sequencing platform, is also frequently used to sequence the transcriptomes of medicinal plants. This platform aims to discover genes and analyze EST-SSRs [53]–[55]. In this study, we sequenced the transcriptome of *L. japonica* buds and leaves using the Roche 454 GS FLX Titanium platform. Our purpose is to discover all candidate genes encoding enzymes and putative transcription factors (TFs) in the CGA and luteoloside biosynthetic pathways to allow for the future synthesis of CGA and luteoloside by heterologous expression in other cell lines.

## Results

### 454 cDNA Sequencing and EST Assembly

**Figure 1:**
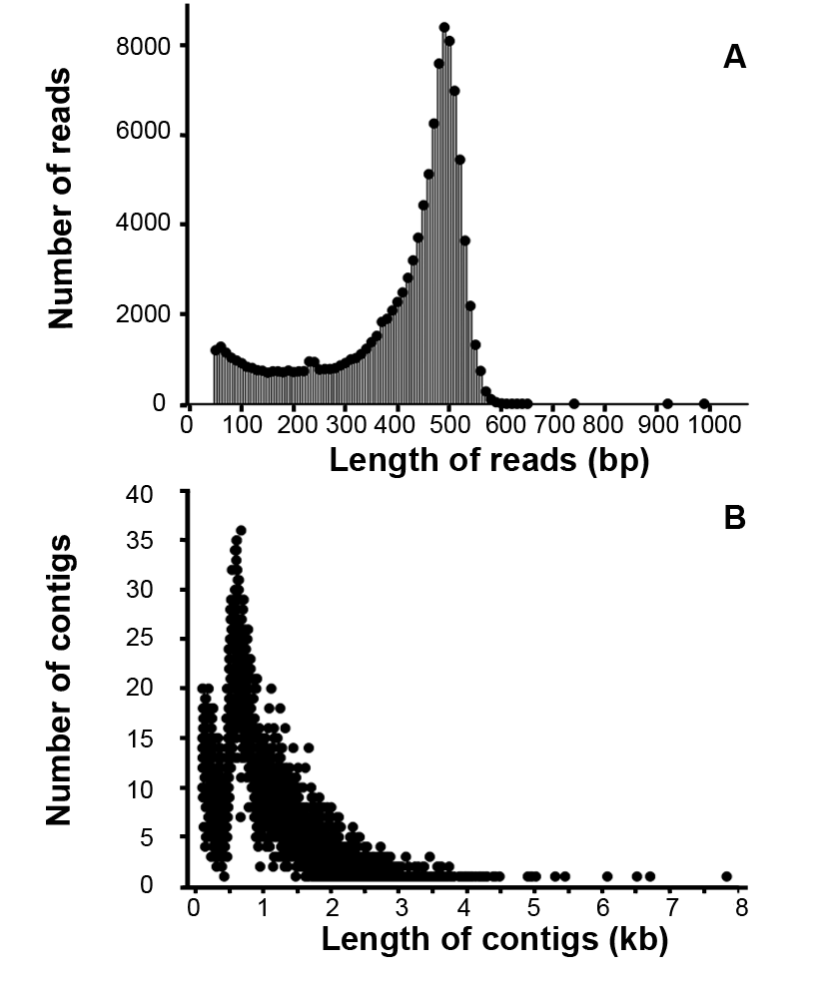
Distribution of the 454 HQ read and contig lengths from *L. japonica*. A. Size distribution of 454 sequencing HQ reads. B. Length distribution of contigs in the EST datasets.

In order to find more genes involved in the biosynthetic pathway of active components, we used buds and leaves for transcriptome sequencing and analysis. Buds are the primary medicinal parts of *L. japonica* [56] and leaves are developing medical resources [57]. Two cDNA libraries were constructed from the total RNA of fresh *L. japonica* buds and leaves using SMART technology. The libraries were subjected to a sequencing run on the 454 GS FLX Titanium platform, each library for half a run. The length distribution of all reads (≥50 bp) of the two libraries can evaluate the quality of the sequence (Figure 1A). After initial quality filtering with the default parameters, the entire run for *L. japonica* buds and leaves produced over one million high-quality (HQ) reads with a total length of 450.9 Mb. The 454-derived ESTs in this study were independently assembled using GS *De Novo* v.2.6 assembler software. After the entire run was assembled, the quality of the assembly (e.g., the ratio of aligned reads, average Contig length, N50 Contig size, largest Contig size) was found to be better than the individual results (Table 1). Overall, 92% of the HQ reads exceeded our minimal quality standards (e.g., SMART primer filtering; length threshold of 50 bp) and were thus used in the assembly. The length distribution of Contigs is shown in Figure 1B. The assembly of the two libraries was used in the following analysis to completely explore their genetic information.

**Table 1:** Summary of *L. japonica* leaves and buds EST sequencing derived from 454 GS FLX Titanium.

	Buds	Leaves	All
**Total bases**	235250941	215654256	450905197
**Total reads**	587840	535470	1123310
**Average reads length**	404	407	406
**Aligned bases**	215517769	204915360	420433129
**Aligned reads**	531719	499708	1031427
**Average Contig length**	1007	879	1127
**N50 Contig Size**	1130	933	1282
**Largest Contig size**	5302	5539	7830
**Number of Contigs**	21863	14382	20940

### Functional Annotation and Categorization

To produce the most informative and complete annotation, all unique sequences were annotated by BLAST [58] against a series of nucleotide and protein databases, including NCBI nucleotide (Nt, released on 03/2012), NCBI non-redundant protein (Nr, released on 03/2012), UniProtKB/SwissProt (released on 03/2012), Kyoto Encyclopedia of Genes and Genomes (KEGG 58) [59], [60], and the *Arabidopsis thaliana* proteome databases (TAIR10) [61]. A total of 64,184 unigenes were compared against these databases with a significance threshold of E-value ≤1e-5. The number of unigenes only can be found in each organ was 32,907 for the buds and 14,566 for the leaves (Figure S1A). Of the 64,184 unigenes, 51,500 (80.24%) unigenes had at least one hit within these databases (Table 2). The remaining unigenes (19.76%) that were not annotated are likely to comprise *L. japonica*-specific genes, as well as genes with homologs in other species, whose corresponding biological functions have not yet been investigated.

**Table 2:** Summary of *L. japonica* leaves and buds EST annotation.

	Total Unigene	Annotated	Percent (%)	Unannotated	Percent (%)
**Nt**	64184	42595	66.4	21589	33.6
**Nr**	64184	47121	73.4	17063	26.6
**Swissprot**	64184	27671	43.1	36513	56.9
**KEGG**	64184	43129	67.2	21055	32.8
**TAIR**	64184	45344	70.6	18840	29.4

**Figure 2:**
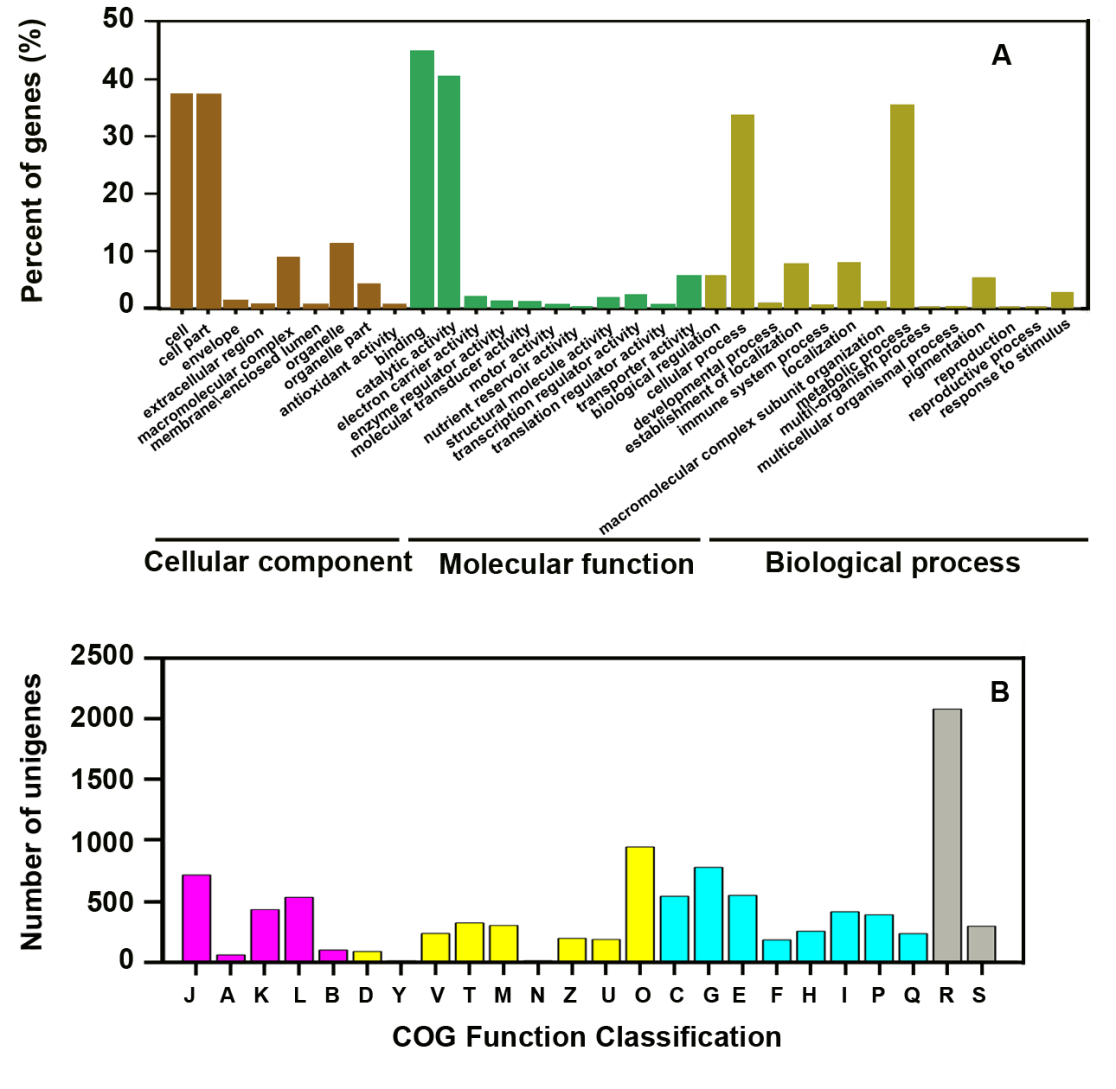
GO and COG classifications of unigenes from *L. japonica*. A. GO function classification of transcriptome. B. COG function classification of transcriptome. Purple boxes represent information storage and processing. J: Translation, ribosomal structure, and biogenesis. A: RNA processing and modification. K: Transcription. L: Replication, recombination and repair. B: Chromatin structure and dynamics. Yellow boxes represent cellular processes and signaling. D: Cell cycle control, cell division, and chromosome partitioning. Y: Nuclear structure. V: Defense mechanisms. T: Signal transduction mechanisms. M: Cell wall/membrane/envelope biogenesis. N: Cell motility. Z: Cytoskeleton. W: Extracellular structures. U: Intracellular trafficking, secretion, and vesicular transport. O: Posttranslational modification, protein turnover, and chaperones. Blue boxes represent metabolism. C: Energy production and conversion. G: Carbohydrate transport and metabolism. E: Amino acid transport and metabolism. F: Nucleotide transport and metabolism. H: Coenzyme transport and metabolism. I: Lipid transport and metabolism. P: Inorganic ion transport and metabolism. Q: Secondary metabolite biosynthesis, transport, and catabolism. Gray boxes represent poorly characterized. R: General function prediction only. S: Function unknown.

The *L. japonica* unigenes were further analyzed and categorized using Gene Ontology (GO) based on the InterPro scan results. GO is an international classification system for standardized gene functions [62]. A total of 19,785 unigenes were classified into three main independent GO categories: molecular function, biological process and cellular component, including 34 subcategories (Figure 2A). Given that a transcript may have multiple hits, some unigenes were classified into two or three categories. The category of biological process, which included 29,623 unigenes, was the largest, followed by the function category (25,306 unigenes) and the cellular location category (16,342 unigenes). Of these categories, protein binding (44.8%) and metabolic process (35.3%) were the two largest subcategories. The percentage of each subcategory in the two organs was shown in Figure S1B. The unigenes only found in the buds or leaves may be related to the development in the two organs. GO analysis showed that the unigenes identified by our sequence run function in various biological processes.

Cluster of orthologous groups (COG) [63] classification is based on comparing protein sequences encoded in complete genomes and presenting major phylogenetic lineages. Only 17.8% (11,414) of the unigenes have been annotated. A total of 9816 of these unigenes clustered into 24 functional categories based on the COG phylogenetic classification (Figure 2B). Among these categories, the “general function prediction only” group was the largest. The “secondary metabolites biosynthesis” group comprised 232 (2.4%) unigenes (Figure 2B). This category includes important factors in the biosynthesis of active secondary metabolites in *L. japonica*.

**Figure 3:**
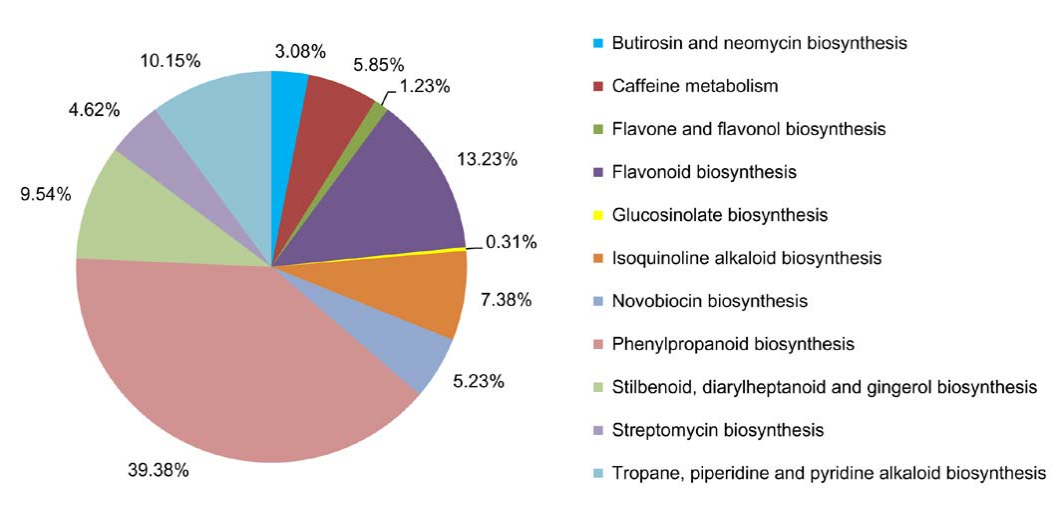
Percentages of *L. japonica* unigenes in 11 subcategories of the metabolic pathway category.

To conduct a more detailed analysis of the unigenes involved in biosynthetic pathways, the unigenes were assigned to the KEGG metabolic pathway category [64]. We mapped 43,129 unigenes to 249 pathways using the KEGG database. Of these unigenes, 9,594 were involved in metabolic pathways and 325 were related to secondary metabolism and mapped to 11 pathways (Figure 3). The phenylpropanoid biosynthesis pathway (ID: ko00940, 39.4%) was the largest group, containing 128 unigenes. Approximately 14.4% of the unigenes were found in the “flavonoid biosynthesis” (ID: ko00941, 13.2%) and “flavone and flavonol biosynthesis” pathways (ID: ko0094). These three categories feature the biosynthesis of the main active compounds, including CGA and luteoloside, in *L. japonica*.

### Discovery of Unigenes Encoding Putative TFs

*L. japonica* has strong adaptability and is widely cultivated in the rock-desertification environment of southwestern China. Some progress has been made in understanding the physiological characteristics of their tolerance to low temperature and drought stress [65]–[67]. However, the molecular mechanism of this resistance remains unclear. Secondary metabolites can protect plants against microbial, herbivore, and ultraviolet attack. The regulation of TFs involved in secondary metabolism in plants is a crucial area of research for exploring plant defense responses in stresses [68], [69]. Various TFs participate in different defense signaling pathways. Thus, the identification of putative TF genes is helpful for realizing the molecular mechanism of *L. japonica* response to environment changes.

In this study, annotation on TAIR was performed to search against the AGRIS (Arabidopsis Gene Regulatory Information Server) [70]. A total of 2,039 unigenes were annotated to TFs in *L. japonica*. These unigenes were annotated in 788 independent coding sequences of *Arabidopsis* TFs that belong to 50 known TF families (Table 3). The zinc finger C2H2 TF family is the most abundant within the *L. japonica* ESTs, including 276 unigenes, followed by the bHLH, C3H, Homeobox, MYB, and AP2/EREBP TF families, which include, 211, 202, 145, 100, and 90 unigenes, respectively, in our data set.

**Table 3:** Top 10 putative transcription factor families in *L. japonica.*

TF family	No. of unigenes	Percent (%)
**C2H2**	276	13.5
**bHLH**	211	10.3
**C3H**	202	9.9
**Homeobox**	145	7.1
**MYB**	100	4.9
**AP2-EREBP**	90	4.4
**GRAS**	81	4.0
**MYB-related**	81	4.0
**Trihelix**	77	3.8
**NAC**	72	3.5

The C2H2, MYB, and AP2/EREBP TF families are important for regulating the response to abiotic stress in plants [71]–[73]. In addition, members of MYB and AP2/EREBP have been found in two plant metabolic pathways, leading to the biosynthesis of phenylpropanoids and terpenoid indole alkaloids, respectively [74]–[76]. The high expression level of TFs in *L. japonica* may be linked to their defense responses and biosynthesis of secondary metabolites. The discovery of these putative TFs in this study may provide useful information for future research.

### Putative Unigenes Encoding Enzymes Involved in the Biosynthesis of CGA and Luteoloside

**Figure 4:**
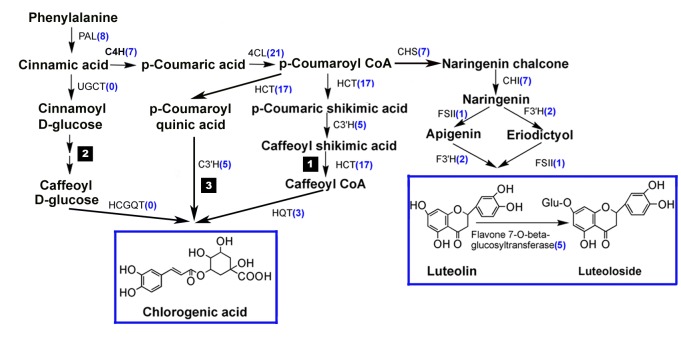
Proposed pathways for the biosynthesis of CGA and luteoloside in *L. japonica*. The three different routes of CGA biosynthesis are labeled 1, 2, and 3. Enzyme names are shown in the pictures. Each enzyme is annotated with the number of corresponding unigenes shown in parentheses. PAL, phenylalanine ammonia lyase; C4H, cinnamate 4-hydroxylase; 4CL, 4-hydroxycinnamoyl CoA ligase/4-coumarate-CoA ligase; HCT, hydroxycinnamoyl CoA shikimate/quinate hydroxycinnamoyltransferase; C3H, *p-*coumarate 3′-hydroxlase; HQT, hydroxycinnamoyl CoA quinate hydroxycinnamoyl transferase; UGCT, UDP glucose: cinnamate glucosyl transferase; HCGQT, hydroxycinnamoyl D-glucose: quinate hydroxycinnamoyl transferase. CHS, chalcone synthesis; CHI, chalcone isomerase; FSII, flavonol synthase; F3'H, Flavonoid 3'-monooxygenase.

CGA and luteoloside are both derived from phenylalanine. We screened the EST dataset and, based on the annotations results, discovered almost all of the enzymes involved in these biosynthetic pathways. Figure 4 shows the number of corresponding unigenes with each enzyme.

The three possible routes of CGA biosynthesis are shown in the order in which they were observed. A total of 61 unigenes were found for CGA biosynthesis (Figure 4, Table S3). Two enzymes that are specific to the second route are not in this EST database. Unigenes encoding *C4H*, *C3′H*, and *HCT* were found in *L. japonica* for the first time. Three unigenes matched *HQT*, one of which is partially *LjHQT* (GQ847546) [38]. HCT and HQT are both hydroxycinnamoyl transferases and their amino acid sequences have high similarity. Twenty unigenes encoding HCT and HQT were all subjected to the following analysis to facilitate the cloning and characterization of *HCT* and *HQT* genes.

Luteolin is normally combined with glucose to form luteoloside in plants. Luteoloside is a type of flavonoid that has an important function in the interaction between plants and their environment. All enzymes involved in luteoloside biosynthesis were found in this study (Table S4). One unigene encoding FSII and two unigenes encoding F3′H were identified in *L. japonica* for the first time. FSII and F3′H belong to the CYP93B and CYP75B families, respectively. Cytochrome P450 (CYP) is a large superfamily that has important functions in the oxidation and hydroxylation reactions in plants. In this study, a total of 188 unique unigenes were annotated as putative CYPs and further classified into 22 families and 30 subfamilies (Table S1). The last step in the biosynthesis of luteoloside is catalyzed by 7-O-beta-glucosyltransferase. Glycosylation can stabilize luteolin in the cells. Glycosyltransferase belongs to another large multigene family in plants. This study found 245 unigenes encoding glycosyltransferase in *L. japonica.* These unigenes are all regarded as important enzymes in many secondary metabolism pathways.

### HQT

**Figure 5:**
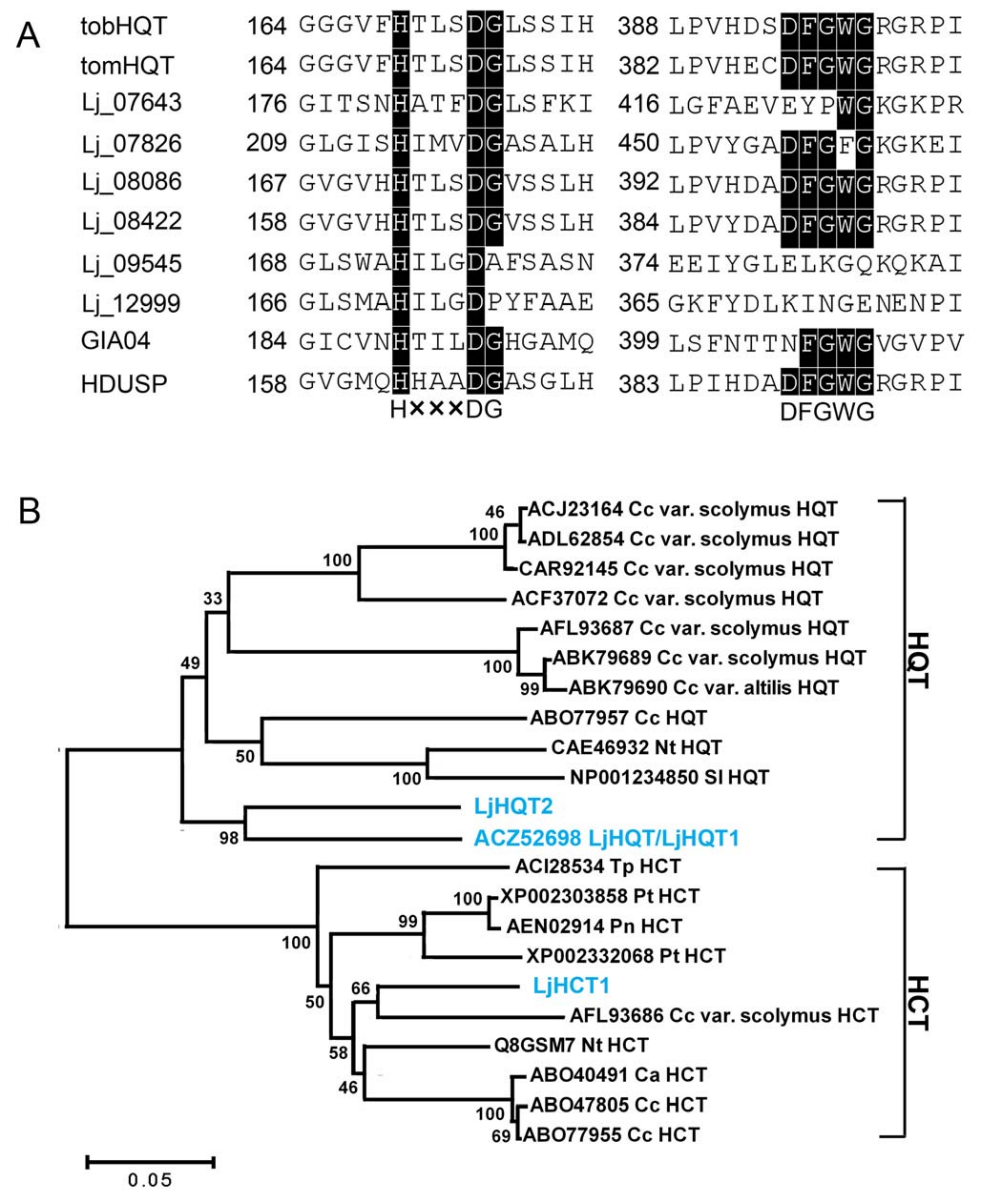
Protein sequence alignment of putative *L. japonica* HQTs and HCTs with representative members of the HQT and HCT families. A. Sequence alignment of the conserved structure motifs in eight *L. japonica* putative HQTs and HCTs with *Solanum lycopersicum* (NP001234850) and *Nicotiana tabacum* (CAE46932). Black boxes indicate the conserved region. B. Neighbor-joining tree of HQTs and HCTs from *L. japonica* and other plants. The HQTs and HCTs used in phylogenetic analysis were retrieved from NCBI, including *Cynara cardunculus* var. scolymus (ACJ23164, ADL62854, CAR92145, ACF37072, AFL93687, ABK79689, ABK79690, AFL93686), *Coffea canephora* (ABO77957, ABO47805, ABO77955), *Nicotiana tabacum* (CAE46932, Q8GSM7), *Solanum lycopersicum* (NP001234850), *Trifolium pratense* (ACI28534), *Populus trichocarpa* (XP002303858, XP002332068), *P. nigra* (AEN02914) and *C. arabica* (ABO40491).

We further analyzed the 17 putative *HCT* unigenes and 3 *HQT* unigenes according to protein domains. In this manner, 10 putative *HQT*/*HCT* genes were identified using bioinformatics analysis. 5′ RACE-PCR and 3′ RACE-PCR were performed according to the unigene sequences in the EST dataset. All of the full-length cDNA sequences of the 10 genes were acquired (Table S2). Among these, singleton HDUSP and BQY55 are two partial sequences of the same gene; contig07826 and singleton H2O5B are two partial sequences of the same gene. We finally obtained 8 ORF sequences and named them: *Lj_07643*, *Lj_07826*, *Lj_08086*, *Lj_08422*, *Lj_09545*, *Lj_12999*, *GIA04,* and *HDUSP* based on their contig and singleton names. These eight sequences were aligned with *Solanum lycopersicum* (NP001234850) and *Nicotiana tabacum* (CAE46932) to identify the conserved regions. The amino acid sequences of Lj_08086, Lj_08422, and HDUSP have the two conserved domains, HXXXDG and DFGWG [39], [77], [78] (Figure 5). Therefore, *Lj_08086*, *Lj_08422*, and *HDUSP* are believed to be putative *HQT* or *HCT* genes in *L. japonica* (Figure 5A).

To further understand the relationship between HQT and HCT in *L. japonica* and the same enzymes in other species, 19 sequences from eight species were retrieved from NCBI. All sequences were divided into two groups, representing HQTs and HCTs in the phylogenetic tree (Figure 5B). HDUSP is believed to be a novel *HCT* gene in *L. japonica* and is named LjHCT1 in this study. In the HCT group, LjHCT1 and *Cynara cardunculus* var. scolymus, which share an identity of 83%, were the most similar (Figure S2).

The amino acid sequence of Lj_08086 is identical to that of LjHQT (ACZ52698) and is named LjHQT1 in this study. *Lj_08422*, a novel *HQT* gene reported in this study, is named *LjHQT2*. LjHQT/LjHQT1 and LjHQT2 were clustered into one sub-group with the highest degree of identity (79%). The shared identity between two HQT proteins in the closed sub-group including *Coffea canephora, N. tabacum* and *S. lycopersicum* ranged from 69% to 72% (Figure S3).

## Discussion

*L. japonica* has pharmacological attributes, including anti-inflammatory and anti-nociceptive properties, and has been believed to be an effective Chinese medicine for thousands of years. Numerous studies have reported the isolation and pharmacological action of the bioactive components in *L. japonica,* including phenolic acids and flavonoids. The potential molecular mechanisms that produce the bioactive biosynthesis of CGA and luteoloside are still not comprehensively understood in *L. japonica*. To gain more genetic information on the secondary metabolites of *L. japonica*, we sequenced two cDNA libraries from *L. japonica* buds and leaves using the Roche/454 GS FLX platform. We identified 51,500 unigenes that we annotated based on Nr, Nt, Swissprot, KEGG, and TAIR, significantly more than that obtained in a previous work. Yuan *et al.* [52] acquired over 32 million reads and over 6000 ESTs from a library made from *L. japonica* buds. They also correlated gene expression profiles to known metabolic activities by comparing them with a transcriptome of a variety (*L. japonica* Thunb. var. chinensis (Watts)) [52]. However, reports describing the key enzymes involved in the biosynthesis pathways of active compounds, such as C4H, C3'H, HCT, and HQT, are unavailable.

We found all of the putative genes encoding the possible enzymes involved in the first and third possible routes of CGA biosynthesis (Figure 4). Regarding the second route, i.e., only one protein, HCGQT, was first purified in the 1970s from sweet potatoes and demonstrated to catalyze caffeoyl-d-glucose and quinic acid to produce CGA *in vitro* [40]–[42]. However, CGA: glucaric acid caffeoyltransferase (CQT) has been found in tomatoes and shown to participate in the transfer of caffeic acid from CGA to glucaric and galactaric acids [79]. Therefore, a route for the recycling of CGA may exist. Whether or not HCGQT is absolutely necessary for the biosynthesis of CGA has yet to be confirmed. Over the past 10 years, the first route involving HQT has attracted the most research attention. *HQT* genes have been discovered in several species, including tobacco, tomato, artichoke, and *L. japonica.* The catalysis of quinic acid and caffeoyl CoA to produce CGA has been identified *in vivo* [35]–[39]. Future experiments to produce CGA through heterologous expression of the two sets of genes in yeast may shed light on the biosynthetic pathway of CGA in *L. japonica*.

Many other related metabolic processes, including carbohydrate metabolic processes as well as amino acid and other secondary metabolic processes, have been mapped based on the KEGG analysis. *L. japonica* contains a large number of volatile constituents that are synthesized through terpenoid metabolism, which may include many CYPs and glycotransferases. In addition, a number of putative TFs have been found in *L. japonica,* some of which are involved in the regulation of metabolic pathway genes. These metabolic pathways and the EST dataset will allow the possibility of intensive studies of *L. japonica*. In addition, the ESTs and unique sequences obtained from this study could provide an important resource for the scientific community interested in the molecular genetics and functional genomics of *L. japonica*.

### Conclusion

We produced one million reads in a whole sequence run using with the Roche/454 GS FLX platform. Based on BLAST, we established an HQ EST database containing 64,184 unigenes for *L. japonica* buds and leaves using 454 GS FLX Titanium sequencing technology. Among these unigenes, 51,500 were annotated and all of the unigenes encoding enzymes involved in the CGA and luteoloside biosynthetic pathways were found. The *HCT* gene in *L. japonica* was cloned for the first time in this study and two *HQT* genes were found in *L. japonica*. In addition, 188 putative CYP unigenes and 245 putative glycosyltransferase unigenes were discovered. In *L. japonica*, *F3'H* and *FSII* encode CYP75B and CYP93B, respectively, which are involved in the biosynthesis of luteoloside. *C3'H* encodes CYP98A, which is involved in the biosynthesis of CGA. CYP may participate in the biosynthesis of triterpenoids in *L. japonica*. Using KEGG analysis, five putative 7-O-beta-glycosyltransferases unigenes that participate in the process of conversion from luteolin to luteoloside were discovered. We initiated a large-scale investigation of the transcriptome of *L. japonica* in terms of functional genomics. A large number of novel putative genes involved in CGA and luteoloside biosynthesis were identified in our EST dataset.

## Materials and Methods

### Plant Materials

*L. japonica* buds and leaves were collected from cultivated fields in Zhengcheng town, Shandong Province, China, in May 2011. The samples used in this study were obtained from local authorities (Linyi Jintai Yaoye Co., Ltd) isolated, rinsed three times with water, snap frozen in liquid nitrogen, and stored at −80°C until RNA isolation.

### RNA Preparation and cDNA Synthesis

Total RNA was isolated using the Universal Plant RNA Isolation Mini Kit (BioTeke, China) according to the manufacturer’s protocol. RNA quality was tested using EtBr-stained 0.8% agarose gels, and the concentration was assessed using a Nanodrop 2000 (Thermo, USA). RNA was treated with TURBO DNase I (Ambion, USA) prior to cDNA synthesis. First-strand cDNA was produced from 1 µg total RNA according to the protocol of the SMARTer™ PCR cDNA Synthesis Kit (Clontech, USA). We used a modified synthetic poly (T) primer (5′-AAG CAG TGG TAT CAA CGC AGA GTG CAG T [20] VN-3′) containing a *Bsg*I digestion site upstream of the poly (T) segment. For double-stranded cDNA synthesis, the cDNA was amplified using the PCR Advantage II polymerase (Clontech) with the following thermal cycle profile: 1 min at 95°C; 17 cycles of 95°C for 15 s, 62°C for 30 s; and finally 68°C for 6 min. The PCR products were purified using the PureLink™ PCR Purification Kit (Invitrogen, USA). The cDNA was then treated overnight with *Bsg*I (NEB, USA) and recovered using the QIAquick PCR Purification Kit (Qiagen, German). Approximately 500 ng of ds cDNA was used for pyrosequencing with the Roche 454 GS FLX Titanium Kit.

### 454 Library Preparation and Sequencing

Approximately 500 ng of amplified cDNA was sheared to produce random fragments of approximately 600 bp to 900 bp. The two libraries of buds and leaves were constructed for Roche 454 sequencing according to the manufacturer’s recommendation. Each library was sent for a 1/2 run using the 454 GS FLX shotgun sequencing platform (454 Life Sciences, Roche).

### EST Assembly

The raw read sequences were processed to screen and filter for weak signals and low-quality reads to yield HQ sequences. The resulting HQ reads were then submitted to the Short Read Archive at NCBI and assigned the accession numbers SRX189761 and SRX189762. The primer and adapter sequences were trimmed from the HQ reads prior to assembly. Sequences shorter than 50 bp were also removed. The remaining HQ ESTs were employed for assembly using the GS FLX *De Novo* Assembly Software, V2.6 (default parameters). After assembly, all sequences, including contigs (obtained from one cluster) and singletons (appeared only once), were named as “unigenes” for subsequent annotation. This Transcriptome Shotgun Assembly project has been deposited at DDBJ/EMBL/GenBank under the accession GAAY00000000. The version described in this paper is the first version, GAAY01000000.

### Annotation and Classification

The unigenes were annotated by a BLAST search against a series of protein and nucleotide databases, including Nr (ftp: //ftp.ncbi.nih.gov/blast/db/FASTA/nr.gz), Nt (ftp: //ftp.ncbi.nih.gov/blast/db/FASTA/nt.gz), Swissprot (http: //www.uniprot.org/downloads), KEGG (ftp: //ftp.genome.jp/pub/kegg/release/archive/kegg/58/), and TAIR (http: //www.arabidopsis.org). The unigenes were compared against these databases with a significance threshold of E-value ≤1e-5. The search was limited to the first five significant hits for each query to maximize computational speed. The definition line of the top BLAST hit was used as a description of the putative function of the queried unigene. Customized Perl scripts were used to parse the BLAST outputs.

### Full-length cDNA Cloning of Putative HQT and HCT Genes

RNA samples of young leaves and buds for gene cloning were converted to first-strand cDNA of the 5' and 3' ends according to the SMART™ RACE cDNA protocol. All genes were amplified at 95°C for 3 min, followed by 25 cycles of 95°C for 30 s, 57°C for 30 s, and 72°C for 1 min 30 s, and a final step at 72°C for 10 min. The recycled products were integrated into a pMD18-T vector (Takara, Dalian, China) and transferred into *Escherichia coli* DH5α competent cells (Transgene, Beijing, China). The isolated clones were sequenced on a 3730XL sequencing platform (ABI, USA).

### Phylogenetic Analysis

All of the hydrocinnamate transferase amino acid sequences were aligned with Clustal X [80] using the following parameters: gap opening penalty, 10; gap extension penalty, 0.1; and delay divergent cutoff, 25%. For the phylogenetic analysis, neighbor-joining tree [81] was constructed under the Jones–Taylor–Thornton substitution model [82] in MEGA4.0 [83] with a bootstrap of 1000 replicates.

## Supporting Information

Figure S1 **The analysis of genes expression in buds and leaves respectively.** A.Venn diagram of the unigenes in the buds and leaves of *L. japonica.* B.Functional classification of unigenes in the two *L. japonica* organs based on GO categories. Unique sequences were classified into three major categories: cellular components, molecular functions and biological processes on the basis of the TAIR GO slim.
(TIF)

Figure S2 **Alignment of HCT amino acid sequence from** ***Lonicera japonica*** **and other species.** *Trifolium pratense* (ACI28534), *Populus trichocarpa* (XP002303858, XP002332068), *Cynara cardunculus* var. scolymus (AFL93686), *Nicotiana tabacum* (Q8GSM7), *Coffea canephora* (ABO77957, ABO47805, and ABO77955). *Lonicera japonica* (LjHCT1).
(TIF)

Figure S3 **Alignment of HQT amino acid sequence from** ***Lonicera japonica*** **and other species.** *Cynara cardunculus* var. scolymus (ACJ23164, ADL62854, CAR92145, ACF37072, AFL93687, ABK79689, and AFL93686), *Coffea canephora* (ABO77957), *Nicotiana tabacum* (CAE46932), *Solanum lycopersicum* (NP001234850). *Lonicera japonica* (ACZ52689/LjHQT1, LjHQT2).
(TIF)

Table S1 **Classification of the candidate CYP genes.**
(DOC)

Table S2 **Summary of the 10 candidate HQT or HCT genes in L. japonica.**
(DOC)

Table S3 **List of putative unigenes related to chlorogenic acid biosynthesis.**
(DOC)

Table S4 **List of putative unigenes related to luteoloside biosynthesis.**
(DOC)
