# Supplementary figures and images for: Transcriptome Analysis of Buds and Leaves Using 454 Pyrosequencing to Discover Genes Associated with the Biosynthesis of Active Ingredients in Lonicera japonica Thunb
Source: PLoS One. 2013 Apr 25;8(4):e62922. doi: 10.1371/journal.pone.0062922 (PMC3636143; doi:10.1371/journal.pone.0062922)

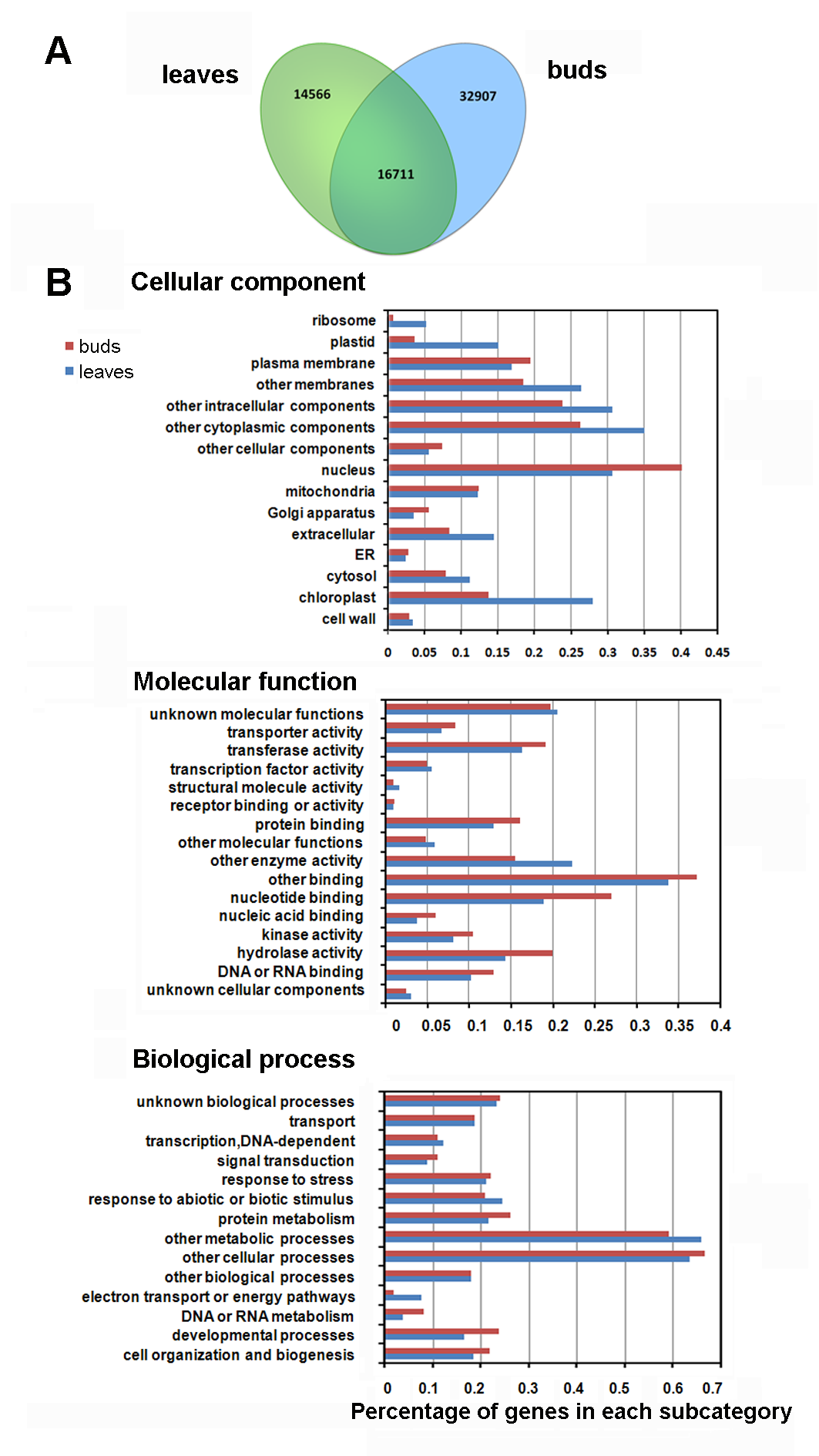

Supplement: Figure S1 — The analysis of genes expression in buds and leaves respectively. A.Venn diagram of the unigenes in the buds and leaves of L. japonica. B.Functional classification of unigenes in the two L. japonica organs based on GO categories. Unique sequences were classified into three major categories: cellular components, molecular functions and biological processes on the basis of the TAIR GO slim. (TIF) [file pone.0062922.s001.tif]

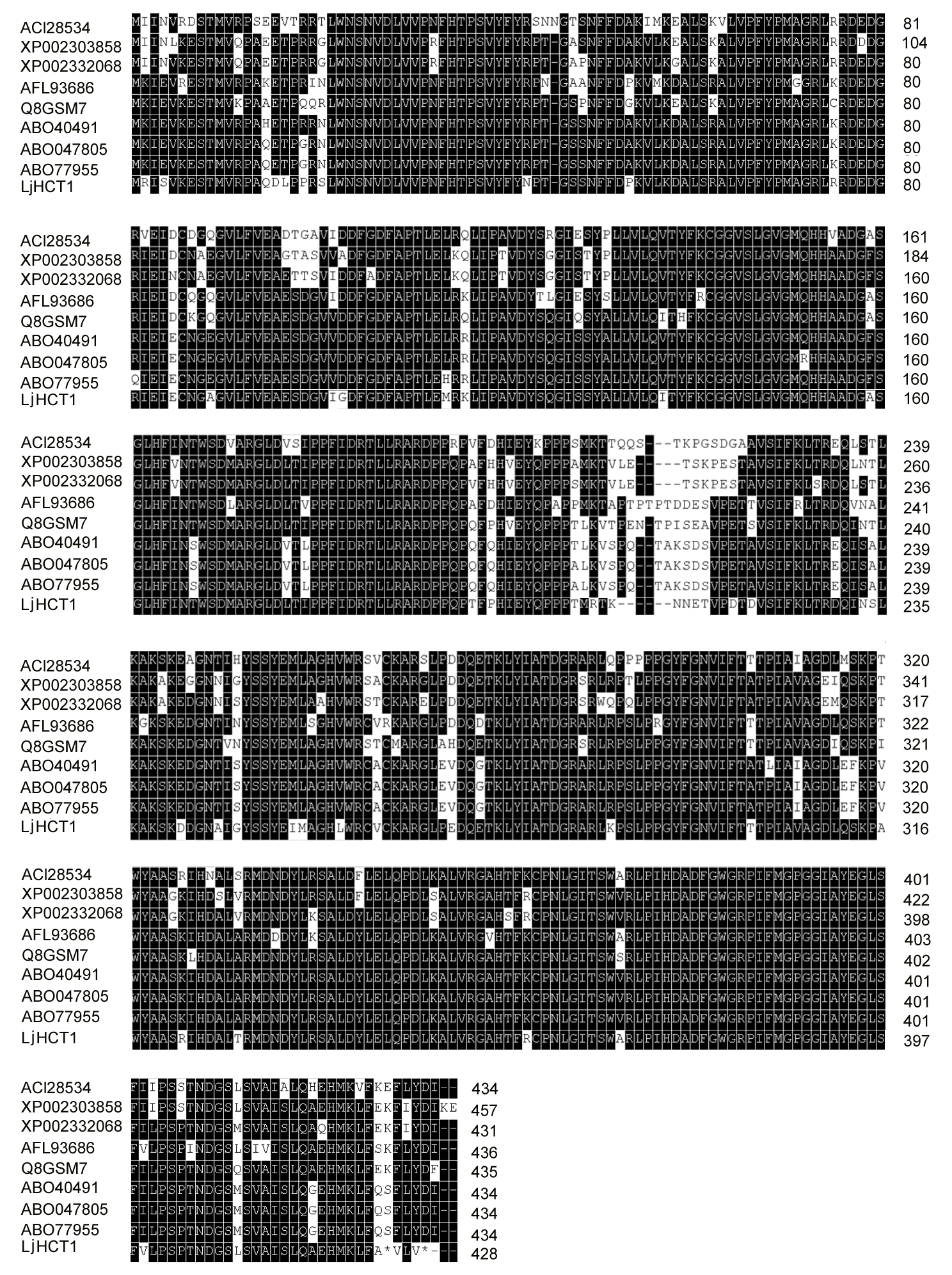

Supplement: Figure S2 — Alignment of HCT amino acid sequence from Lonicera japonica and other species. Trifolium pratense (ACI28534), Populus trichocarpa (XP002303858, XP002332068), Cynara cardunculus var. scolymus (AFL93686), Nicotiana tabacum (Q8GSM7), Coffea canephora (ABO77957, ABO47805, and ABO77955). Lonicera japonica (LjHCT1). (TIF) [file pone.0062922.s002.tif]

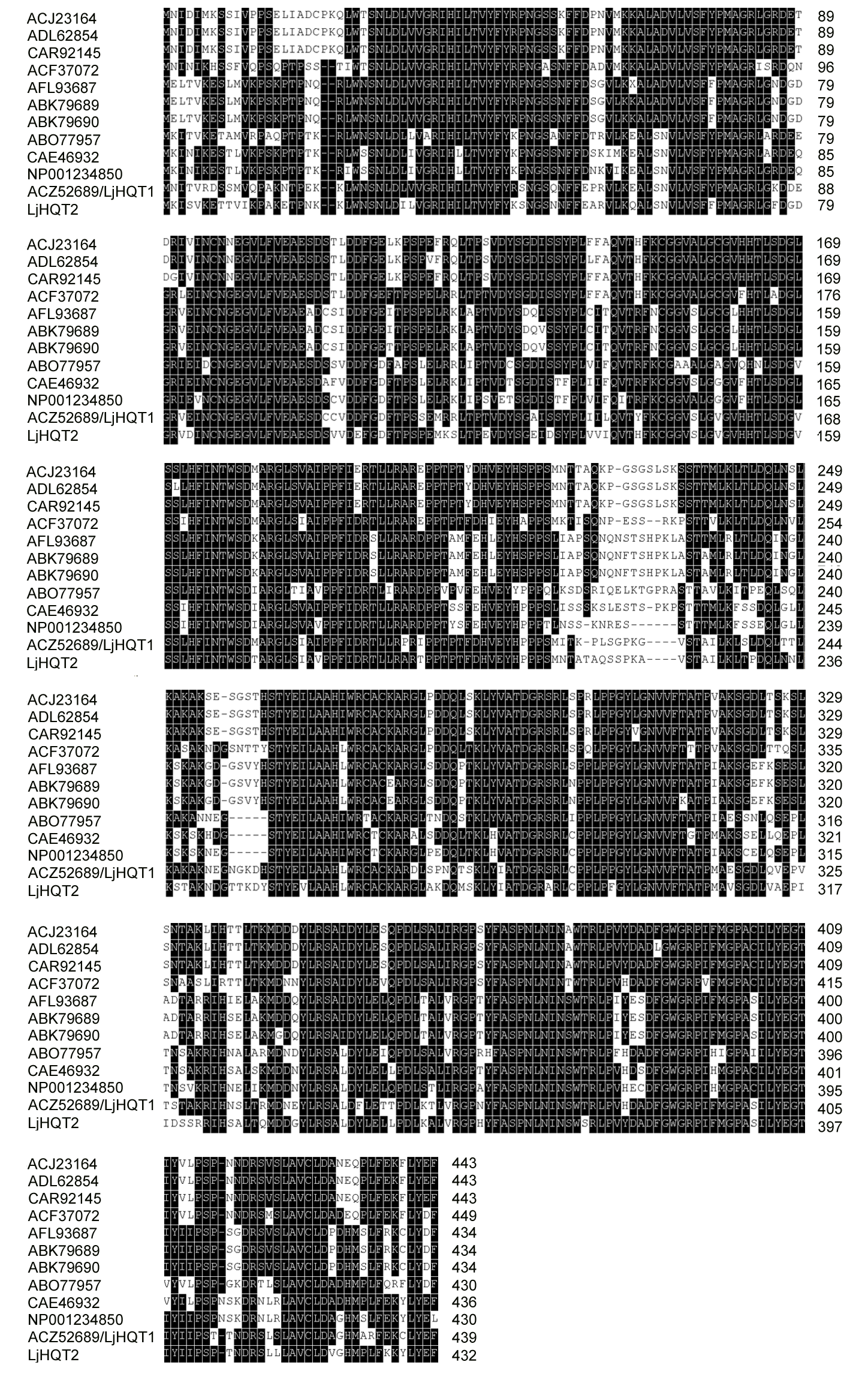

Supplement: Figure S3 — Alignment of HQT amino acid sequence from Lonicera japonica and other species. Cynara cardunculus var. scolymus (ACJ23164, ADL62854, CAR92145, ACF37072, AFL93687, ABK79689, and AFL93686), Coffea canephora (ABO77957), Nicotiana tabacum (CAE46932), Solanum lycopersicum (NP001234850). Lonicera japonica (ACZ52689/LjHQT1, LjHQT2). (TIF) [file pone.0062922.s003.tif]
