# Supplementary material for: Transcriptome Analysis of Buds and Leaves Using 454 Pyrosequencing to Discover Genes Associated with the Biosynthesis of Active Ingredients in Lonicera japonica Thunb
Source: PLoS One. 2013 Apr 25;8(4):e62922. doi: 10.1371/journal.pone.0062922 (PMC3636143; doi:10.1371/journal.pone.0062922)
Supplement: Table S1 — Classification of the candidate CYP genes. (DOC) [file pone.0062922.s004.doc]

**Table S1 Classification of the candidate CYP** genes

|  | **number of subfamily** | **number of unigene** |
| --- | --- | --- |
| CYP705 | 1 | 3 |
| CYP710 | 1 | 1 |
| CYP716 | 1 | 12 |
| CYP71 | 3 | 34 |
| CYP72 | 1 | 3 |
| CYP734 | 1 | 19 |
| CYP750 | 1 | 1 |
| CYP76 | 2 | 13 |
| CYP77 | 1 | 6 |
| CYP78 | 1 | 9 |
| CYP81 | 2 | 11 |
| CYP82 | 3 | 18 |
| CYP83 | 1 | 1 |
| CYP84 | 1 | 4 |
| CYP85 | 1 | 4 |
| CYP86 | 2 | 22 |
| CYP89 | 1 | 2 |
| CYP90 | 2 | 2 |
| CYP93 | 1 | 2 |
| CYP94 | 1 | 10 |
| CYP97 | 1 | 1 |
| CYP98 | 1 | 5 |
| unclassified CYP | 0 | 5 |
| total | 30 | 188 |
