# Supplementary material for: Transcriptome Analysis of Buds and Leaves Using 454 Pyrosequencing to Discover Genes Associated with the Biosynthesis of Active Ingredients in Lonicera japonica Thunb
Source: PLoS One. 2013 Apr 25;8(4):e62922. doi: 10.1371/journal.pone.0062922 (PMC3636143; doi:10.1371/journal.pone.0062922)
Supplement: Table S2 — Summary of the 10 candidate HQT or HCT genes in L. japonica. (DOC) [file pone.0062922.s005.doc]

**Table S2 Summary of the 10 candidate HQT or HCT genes in *L. japonica***

| No. | EST name | Gene Name | Length(bp) |
| --- | --- | --- | --- |
| 1 | Contig08086 | *Lj_08086/LjHQT1* | 1320 |
| 2 | Contig08422 | *Lj_08422/LjHQT2* | 1296 |
| 3 | HDUSP/BQY55 | *HDUSP/LjHCT1* | 1275 |
| 4 | Contig07826/H2O5B | *Lj_07826* | 1497 |
| 5 | Contig07643 | *Lj_07643* | 1410 |
| 6 | Contig12999 | *Lj_12999* | 1338 |
| 7 | Contig09545 | *Lj_09545* | 1284 |
| 8 | GIA04 | *GIA04* | 1302 |
