# Supplementary material for: Transcriptome Analysis of Buds and Leaves Using 454 Pyrosequencing to Discover Genes Associated with the Biosynthesis of Active Ingredients in Lonicera japonica Thunb
Source: PLoS One. 2013 Apr 25;8(4):e62922. doi: 10.1371/journal.pone.0062922 (PMC3636143; doi:10.1371/journal.pone.0062922)
Supplement: Table S3 — List of putative unigenes related to chlorogenic acid biosynthesis. (DOC) [file pone.0062922.s006.doc]

**Table S3. List of putative unigenes related to chlorogenic acid biosynthesis**.

| **EST ID** | **E-value** | **Identity (%)** | **Annotation (BlastX)** |
| --- | --- | --- | --- |
| **PAL: phenylalanine ammonia lyase** | | | |
| contig06879 | 0.0 | 91 | PREDICTED: phenylalanine ammonia-lyase [*Vitis vinifera*] |
| contig09227 | 0.0 | 88 | PREDICTED: phenylalanine ammonia-lyase [*Vitis vinifera*] |
| contig09420 | 0.0 | 89 | phenylalanine ammonia-lyase [*Daucus carota*] |
| contig12583 | 4E-163 | 89 | phenylalanine ammonia lyase 2 [*Platycodon grandiflorus*] |
| contig14703 | 0.0 | 94 | phenylalanine ammonia lyase 2 [*Platycodon grandiflorus*] |
| contig15070 | 1E-96 | 84 | phenylalanine ammonia lyase [*Robinia pseudoacacia*] |
| G5N1V3W01EP7LD | 5E-20 | 75 | phenylalanine ammonia-lyase [*Lactuca sativa*] |
| G5N1V3W02GYASR | 4E-55 | 90 | phenylalanine ammonia lyase [*Catharanthus roseus*] |
| **C4H: cinnamate 4-hydroxylase** | | | |
| contig12924 | 0.0 | 92 | cinnamate 4-hydroxylase, putative [*Ricinus communis*] |
| contig12926 | 0.0 | 93 | cinnamate-4-hydroxylase [*Gossypium hirsutum*] |
| contig20312 | 1E-66 | 96 | cinnamate 4-hydroxylase, partial [*Populus alba*] |
| contig20357 | 2E-85 | 93 | cinnamate 4-hydroxylase [*Acacia mangium*] |
| G5N1V3W02HY8WK | 7E-72 | 88 | PREDICTED: trans-cinnamate 4-monooxygenase [*Vitis vinifera*] |
| G5N1V3W02HYM2P | 7E-43 | 81 | PREDICTED: trans-cinnamate 4-monooxygenase [*Vitis vinifera*] |
| G5N1V3W02JIEXG | 1E-67 | 88 | PREDICTED: trans-cinnamate 4-monooxygenase [*Vitis vinifera*] |
| **4CL: 4-hydroxycinnamoyl CoA ligase** | | | |
| contig07375 | 0.0 | 66 | PREDICTED: 4-coumarate--CoA ligase-like 9-like [*Glycine max*] |
| contig07436 | 0.0 | 84 | 4-coumarate:coenzyme A ligase [*Nicotiana tabacum*] |
| contig07538 | 0.0 | 68 | 4-coumarate-coa ligase [*Populus trichocarpa*] |
| contig08624 | 0.0 | 87 | PREDICTED: putative peroxisomal-coenzyme A synthetase-like [*Vitis vinifera*] |
| contig10662 | 6E-133 | 86 | 4-coumarate--CoA ligase-like 7-like [*Glycine max*] |
| contig10801 | 2E-163 | 81 | 4-coumarate--CoA ligase-like protein [*Arabidopsis thaliana*] |
| contig11274 | 1E-123 | 62 | PREDICTED: 4-coumarate--CoA ligase-like 9 [*Vitis vinifera*] |
| contig12253 | 3E-120 | 68 | PREDICTED: 4-coumarate--CoA ligase-like 7 [*Vitis vinifera*] |
| contig14294 | 7E-110 | 73 | PREDICTED: 4-coumarate--CoA ligase-like 5 [*Vitis vinifera*] |
| contig15261 | 2E-102 | 74 | PREDICTED: 4-coumarate--CoA ligase-like 9 [*Vitis vinifera*] |
| contig19005 | 6E-88 | 74 | 4-coumarate-CoA ligase [*Medicago truncatula*] |
| G5N1V3W01B5SMX | 7E-29 | 77 | 4-coumarate-CoA ligase-like protein [*Arabidopsis thaliana*] |
| G5N1V3W02F3V8Q | 1E-34 | 81 | PREDICTED: 4-coumarate--CoA ligase-like 5 [*Vitis vinifera*] |
| G5N1V3W02GI1DX | 1E-51 | 79 | 4-coumarate:CoA ligase-like [*Nicotiana sylvestris*] |
| G5N1V3W02G96X6 | 3E-28 | 75 | 4-hydroxycinnamoyl-CoA ligase 2 [*Coffea arabica*] |
| G5N1V3W02H34XF | 1E-72 | 77 | 4-coumarate:CoA ligase-like [*Nicotiana sylvestris*] |
| G5N1V3W02H5JI0 | 3E-80 | 86 | 4-coumarate:CoA ligase-like [*Nicotiana sylvestris*] |
| G5N1V3W02HSV1I | 5E-28 | 75 | 4-hydroxycinnamoyl-CoA ligase 2 [*Coffea arabica*] |
| G5N1V3W02JFRCQ | 8E-98 | 82 | 4-coumarate-CoA ligase-like protein [*Arabidopsis thaliana*] |
| G5N1V3W02HPS2W | 1E-27 | 64 | 4-coumarate-CoA ligase-like protein [*Arabidopsis thaliana*] |
| G5N1V3W02JDB0N | 5E-44 | 72 | PREDICTED: 4-coumarate--CoA ligase-like 5 [*Vitis vinifera*] |
| **CYP98A3/C3’H: p-coumarate 3’-hydroxlase** | | | |
| contig07175 | 0 | 77 | p-coumaroyl quinate/shikimate 3'-hydroxylase [*Lonicera japonica*] |
| contig12928 | 0 | 91 | p-coumarate 3'-hydroxylase [*Platycodon grandiflorus*] |
| *contig13559 | 3E-45 | 83 | cytochrome P450 (CYP98A27) [*Populus trichocarpa*] (BLAST in Nt database) |
| G5N1V3W01BBE5D | 3E-44 | 64 | CYP92A46 [*Scoparia dulcis*] |
| G5N1V3W02IRGX5 | 3E-73 | 91 | p-coumaroyl-shikimate 3'-hydroxylase [*Trifolium pratense*] |
| **HQT/HCT: hydroxycinnamoyl CoA shikimate/quinate hydroxycinnamoyltransferase** | | | |
| contig01728 | 1E-15 | 50 | AB10949.1| anthranilate N-hydroxycinnamoyl/benzoyltransferase-like protein [Arabidopsis thaliana] gb|AAL36423.1| putative anthranilate N-hydroxycinnamoyl/benzoyltransferase [*Arabidopsis thaliana*] gb|AAM51419.1| putative anthranilate N-hydroxycinnamoyl/benzoyltransferase [Arabidopsis thaliana] gb|AAM61217.1| anthranilate N-hydroxycinnamoyl/benzoyltransferase-like protein [Arabidopsis thaliana] gb|AED98307.1| HXXXD-type acyl-transferase-like protein [Arabidopsis thaliana] |
| contig07625 | 2E-168 | 56 | PREDICTED: hydroxycinnamoyl-Coenzyme A shikimate/quinate hydroxycinnamoyltransferase [*Vitis vinifera*] |
| contig07643 | 1e-171 | 65 | PREDICTED: omega-hydroxypalmitate O-feruloyl transferase [Vitis vinifera] emb|CBI38342.3| unnamed protein product [Vitis vinifera] |
| contig07826 | 0.0 | 62 | hydroxycinnamoyl-Coenzyme A shikimate/quinate hydroxycinnamoyltransferase-like [*Vitis vinifera*] |
| contig08422 | 0.0 | 79 | Hydroxycinnamoyl-Coenzyme A shikimate/quinate hydroxycinnamoyltransferase [*Lonicera japonica*] |
| contig09545 | 6e-157 | 54 | PREDICTED: hydroxycinnamoyl-Coenzyme A shikimate/quinate hydroxycinnamoyltransferase [*Vitis vinifera*] |
| *contig12999 | 6e-10 | 84 | PREDICTED: Vitis vinifera hydroxycinnamoyl-Coenzyme A shikimate/quinate hydroxycinnamoyltransferase-like (LOC100252165), mRNA |
| G5N1V3W01ANINV | 4E-09 | 75 | PREDICTED: hydroxycinnamoyl-Coenzyme A  shikimate/quinate hydroxycinnamoyltransferase-like [*Vitis vinifera*] |
| G5N1V3W01BQY55 | 1E-76 | 90 | hydroxycinnamoyl-CoA shikimate/quinate hydroxycinnamoyl transferase [*Coffea arabica*] |
| G5N1V3W01CW5FT | 5E-12 | 51 | PREDICTED: hydroxycinnamoyl-Coenzyme A  shikimate/quinate hydroxycinnamoyltransferase-like [*Vitis vinifera*] |
| G5N1V3W01DLWXU | 2E-67 | 92 | Hydroxycinnamoyl-Coenzyme A shikimate/quinate hydroxycinnamoyltransferase [*Nicotiana tabacum*] |
| G5N1V3W01E1RCK | 5E-51 | 60 | PREDICTED: hydroxycinnamoyl-Coenzyme A  shikimate/quinate hydroxycinnamoyltransferase [*Vitis vinifera*] |
| G5N1V3W02GDPHG | 3E-53 | 89 | hydroxycinnamoyl-CoA shikimate/quinate hydroxycinnamoyl transferase [*Coffea arabica*] |
| G5N1V3W02GIA04 | 3E-67 | 58 | hydroxycinnamoyl-CoA shikimate/quinate hydroxycinnamoyl transferase [*Coffea arabica*] |
| G5N1V3W02GKWC0 | 2E-105 | 99 | hydroxycinnamoyl-CoA shikimate/quinate hydroxycinnamoyltransferase [*Lonicera japonica*] |
| G5N1V3W02H8IQD | 3E-24 | 58 | hydroxycinnamoyl-CoA shikimate/quinate hydroxycinnamoyl transferase [*Coffea arabica*] |
| G5N1V3W02HXAAT | 8E-78 | 91 | hydroxycinnamoyl-CoA shikimate/quinate hydroxycinnamoyltransferase [*Coffea arabica*] |
| **HQT: hydroxycinnamoyl CoA quinate hydroxycinnamoyl transferase** | | | |
| contig08086 | 0.0 | 100 | hydroxycinnamoyl-CoA quinate hydroxycinnamoyltransferase [*Lonicera japonica*] |
| G5N1V3W01BFCYJ | 2E-34 | 70 | hydroxcinnamoyl-CoA quinate/shikimate hydroxycinnamoyltransferase,  partial [*Populus alba*] |
| G5N1V3W02HDUSP | 1E-103 | 91 | hydroxycinnamoyl-CoA:quinate hydroxycinnamoyltransferase [*Cynara cardunculus*] |

*All the annotation information of the unigenes comes from Nr hit. The description of contig13559 and contig12999 come from Nt hit.
