# Supplementary material for: Transcriptome Analysis of Buds and Leaves Using 454 Pyrosequencing to Discover Genes Associated with the Biosynthesis of Active Ingredients in Lonicera japonica Thunb
Source: PLoS One. 2013 Apr 25;8(4):e62922. doi: 10.1371/journal.pone.0062922 (PMC3636143; doi:10.1371/journal.pone.0062922)
Supplement: Table S4 — List of putative unigenes related to luteoloside biosynthesis. (DOC) [file pone.0062922.s007.doc]

**Table S4. List of putative unigenes related to luteoloside biosynthesis.**

| **EST ID** | **E value** | **Identity (%)** | **Annotation (BlastX)** |
| --- | --- | --- | --- |
| **CHI: chalcone isomerase** | | | |
| contig11653 | 3E-138 | 97 | chalcone isomerase, partial [*Lonicera japonica*] |
| contig20209 | 5E-33 | 81 | PREDICTED: chalcone--flavonone isomerase-like isoform 2 [*Vitis vinifera*] |
| contig20579 | 1E-33 | 86 | chalcone isomerase [*Gossypium hirsutum*] |
| G5N1V3W02IBI28 | 8E-51 | 74 | chalcone isomerase-like protein [*Populus trichocarpa*] |
| G5N1V3W02H6XNR | 2E-60 | 78 | chalcone isomerase-like protein [*Populus trichocarpa*] |
| G5N1V3W02I6WQ8 | 3E-38 | 83 | chalcone isomerase-like protein [*Populus trichocarpa*] |
| G5N1V3W01EJUPC | 2E-59 | 80 | chalcone isomerase-like protein [*Populus trichocarpa*] |
| **CHS: chalcone synthesis** | | | |
| contig04178 | 2E-170 | 94 | chalcone synthase [*Vitis vinifera*] |
| contig15325 | 4E-151 | 93 | naringenin-chalcone synthase [*Juglans nigra x Juglans regia*] |
| contig17273 | 2E-110 | 98 | chalcone synthase [*Lonicera japonica*] |
| contig19165 | 2E-72 | 86 | chalcone synthase [*Lilium hybrid division VII*] |
| G5N1V3W02IEDRY | 1E-55 | 93 | chalcone synthase [*Juglans nigra*] |
| G5N1V3W02I74IZ | 1E-66 | 76 | chalcone synthase [*Petunia x hybrida*] |
| G5N1V3W02F7P7U | 6E-53 | 69 | PREDICTED: chalcone synthase A [*Vitis vinifera*] |
| **F3H: p-coumarate 3-hydroxlase** | | | |
| contig18760 | 2E-83 | 67 | cytochrome P450 CYP736A54 [*Bupleurum chinense*] |
| G5N1V3W01C7PUH | 1E-51 | 66 | flavonoid 3-hydroxylase, putative [*Ricinus communis*] |
| **FSⅡ: flavonol synthase** | | | |
| contig08285 | 0.0 | 69 | flavone synthase II [*Lobelia erinus*] |
| **F3'H: Flavonoid 3'-monooxygenase** | | | |
| contig08352 | 0.0 | 79 | PREDICTED: flavonoid 3'-monooxygenase [*Vitis vinifera*] |
| contig06963 | 0.0 | 74 | PREDICTED: cytochrome P450 93A1-like [*Vitis vinifera*] |
| **GT: Glycosyltransferase (flavone 7-O-beta-glucosyltransferase)** | | | |
| contig14791 | 7E-76 | 55 | UDP-glucose:flavonoid 7-O-glucosyltransferase mRNA, complete cds [*Malus x domestica*] |
| contig03762 | 1E-88 | 62 | UDP-glucose:flavonoid 7-O-glucosyltransferase [*Malus x domestica*] |
| G5N1V3W02I3LCL | 5E-38 | 54 | UDP-glucose:flavonoid 7-O-glucosyltransferase mRNA, complete cds [*Malus x domestica*] |
| G5N1V3W02HB6YK | 2E-34 | 59 | UDP-glucose:flavonoid 7-O-glucosyltransferase [*Malus x domestica*] |
| G5N1V3W02F74VQ | 5E-21 | 67 | UDP-glucose:flavonoid 7-O-glucosyltransferase mRNA, complete cds [*Malus x domestica*] |
